# Physical Activity Recommendations for Segments of School Days in Adolescents: Support for Health Behavior in Secondary Schools

**DOI:** 10.3389/fpubh.2020.527442

**Published:** 2020-10-30

**Authors:** Karel Frömel, Dorota Groffik, Josef Mitáš, Andrea Madarasová Gecková, Tamás Csányi

**Affiliations:** ^1^Faculty of Physical Culture, Palacký University, Olomouc, Czechia; ^2^Institute of Sport Science, The Jerzy Kukuczka Academy of Physical Education, Katowice, Poland; ^3^Faculty of Medicine, Pavol Jozef Šafárik University in Košice, Košice, Slovakia; ^4^Department of Physical Education, Faculty of Primary and Pre-School Education, ELTE Eötvös Loránd University, Budapest, Hungary; ^5^Health Enhancing Physical Activity Department, Hungarian School Sport Federation, Budapest, Hungary

**Keywords:** physical education, steps, physical literacy, school lifestyle, comprehensive school physical activity program

## Abstract

School physical activity (PA) is an indispensable part of daily PA, the foundation for developing lifelong PA and fitness, and an easy way to gain physical and health literacy. School PA is equally important for understanding the continuity of physical and mental health, even in broader psychosocial aspects. Regarding long-term outcomes, significant attention has been paid to the determination of daily and weekly recommendations for adolescent PA. However, comprehensive approaches suggesting recommendations for PA in segments of the school day are rare. This study aimed to (a) provide a rationale for PA recommendations in segments of school days and incorporate it into generally accepted PA recommendations, and (b) promote radical changes in the educational process toward a healthy school lifestyle through PA recommendations in segments of school days. The results of research conducted in 98 secondary schools in the Czech Republic and 104 secondary schools in Poland from 2009 to 2017 were used in this study. In total, 3,860 boys and 5,237 girls from the Czech Republic and 3,052 boys and 3,329 girls from Poland, all aged 15–19, participated. We recommended at least 2,000 steps (or 10 min of moderate-to-vigorous PA) for the before school segment and at least 6,000 steps (or 30 min of moderate-to-vigorous PA) for the after-school segment. For the time spent at school, we further recommended at least 500 steps/h (alternatively, at least 3,000 steps/school time), 20 min of moderate-to-vigorous PA (≥3METs or 60% HRmax), and at least one HRsubmax/max response to significant stress during PA to mitigate educational stress and high levels of sedentary behavior in schools. PA should account for at least 25% of school time, even on days with no scheduled physical education lessons. We propose using PA recommendations in segments of school days to achieve positive changes in the educational process and school lifestyle. Acceptance of PA recommendations in segments of school days theories on physical education could help refine and concretize demands for changes in PA and lifestyle in secondary schools. In practice, it could support the creation of innovative and comprehensive school PA programs. Future research should focus on obtaining evidence in support for adolescent PA by applying PA recommendations in segments of school days.

## Introduction

The state of health in European populations is alarming, most notably with cardiovascular diseases being responsible for 45% (3.9 million) of all deaths in Europe annually (1). Though progressive improvement has been observed in some European countries, the situation in Central and Eastern Europe remains relatively unchanged. As children age, rates of overweight and obesity rise (2), cardiovascular fitness declines (3), incorrect body posture increases (4), and mental health disorders increase, especially among girls (5). There is also a decline in physical activity (PA) as children age (6, 7), particularly for intense PA (8), along with decreasing interest in physical education (PE) (9). Indeed, by 2012, only 35 of 131 European studies reported that adolescents met daily PA recommendations (10) and in Central-Eastern European countries, high levels of sedentary behaviors have been recorded (11). Schools bear some responsibility for this situation, as they should significantly contribute to health maintenance, developing physical and mental fitness, and promote healthy lifestyles for adolescents.

The school environment is the ideal “practical lab” to investigate the effects of education on adolescent health. In particular, schools play a crucial role in promoting PA, as well as the adoption of lifelong PA habits (12); this is especially true for combating sedentary behavior in and out of school (13), and identifying and supporting adolescents with low physical fitness (14). Numerous school reforms and emerging alternative education systems seek positive changes in adolescent health (15), including the implementation of PA recommendations in different segments of school days; we defined segments of school days as the time before, during, and after school that is set aside for PA on days that adolescents attend school. However, there is legitimate criticism of the limited evidence-base for current PA recommendations (16). The question arises whether universal PA recommendations in segments of school days can be formulated to transcend differences in geographical, urban, socioeconomic, and, in particular, educational conditions.

Thus, this study aimed to:

(1) Through the synthesis of previous research results, provide a rationale for PA recommendations in segments of school days and incorporate it into generally accepted PA recommendations.
(2) Promote a comprehensive approach to change educational processes toward a healthy school lifestyle for adolescents through PA recommendations in segments of school days.

## Materials and Methods

### Methodological Basis

This study's methodological basis is rooted in analyzing investigations of school health promotion models (17), together with recommendations for PA for children and adolescents (18, 19), school PA, and PE (20, 21). This study is based on a synthesis of previous research published in this area. Specifically, it draws on research findings from studies conducted in secondary schools in the Czech Republic and Poland (22–29), where the methodology of individual research is clearly presented; therefore, it is not repeated in this comprehensive study.

### Participants and Procedure

From 2009 to 2017, we conducted surveys on PA and inactivity among adolescents between the ages of 15 and 19 in 98 secondary schools in the Czech Republic (3,860 boys and 5,237 girls) and 104 secondary schools in Poland (3,052 boys and 3,329 girls). Subsets of school participants had an average age 16–17 years with BMI ranging from 20 to 22 kg·m^−2^ and only 5–10% of adolescents did not give informed consent to the research. All participants received individual feedback on the research results and the average results were discussed at the end of the research. This study presents the results directly related to school PA from a range of measures: the International Physical Activity Questionnaire–Long Form [IPAQ-LF; (30)], weekly PA monitoring using pedometers, and daily PA monitoring using accelerometers. The IPAQ-LF was completed by 1,632 boys and 2,537 girls to estimate perceived PA in selected domains; evaluating the school PA domain was the focus of this study [see (31–34)]. In the Czech version of the IPAQ-LF the Pearson's correlation coefficient, as an indicator of concurrent validity between weekly PA (METs-min) and weekly step count (steps/week), was *r* = 0.283. The internal consistency reliability coefficient Cronbach's alpha was α = 0.845. There were 810 boys and 1,293 girls who participated in weekly PA monitoring using Digi-Walker SW-700 pedometers [Yamax Co., Yasama Corp., Tokyo, Japan; see (22, 33, 34)], and 1,136 boys and 2,256 girls who participated in monitoring daily school PA using ActiTrainer accelerometers (Pensacola, FL, USA; http://www.theactigraph.com/products/actitrainer). Accelerometer data were processed using cutoff points for children (35), and the epoch was set at 15s [see (22, 23, 26)]. We set the threshold for the moderate-to-vigorous PA (MVPA) in accordance with the recommendations of Norton et al. (36), All surveys were centrally managed and conducted by stable research teams in both countries.

We respect the specifics of contemporary education in Central-Eastern European countries, particularly the frequent, and not always system-based, educational reforms resulting from extensive political changes. In these countries, the educational system tends to prefer imparting cognitive knowledge to students; consequently, the relationship between sports and PE tends to be separated, and PE lessons are typically sex-segregated.

Data collection, recording, and evaluation were performed using the respective Czech and Polish versions of the International Database for Research and Educational Support (Indares; www.indares.com).

### Statistical Analysis

Statistical analyses were conducted using SPSS 22 (IBM Corp., Armonk, NY) and Statistica 13 (StatSoft Inc., Prague, Czech 320 Republic). For statistical processing, we used descriptive characteristics and cross-tables to identify differences in meeting PA recommendations for boys and girls. A Kruskal-Wallis analysis of variance (ANOVA) was applied for IPAQ-LF results, and one or two-way ANOVAs with Scheffé *post-hoc* analyses were used for the analysis of data obtained from monitoring PA with pedometers and accelerometers. The practical significance of the results was assessed by partial eta-squared ($\eta_{p}^{2}$) and Cohen's *w* effect size coefficients, defined as follows: small 0.01 ≤ $\eta_{\text{p}}^{2}$ < 0.06 (0.10 ≤ w < 0.29), medium 0.06 ≤ $\eta_{\text{p}}^{2}$ < 0.14 (0.3 ≤ w < 0.50), and large $\eta_{\text{p}}^{2}$ ≥ 0.14 (w ≥ 0.50). Statistical significance was set at *p* < 0.05.

### Ethics Statement

The study was approved by the Ethics Committee of Human Research of the Faculty of Physical Culture, Palacký University, in Olomouc (no. 24/2012) and the Jerzy Kukuczka Academy of Physical Education, in Katowice (no. 2/2008). All participants, their parents, and school administrators provided written informed consent. Prior to the study, the participants received detailed information on data confidentiality and security in Indares, as well as on data processing and publishing. Participants could withdraw from PA monitoring at any time during the study.

## Results

### General Recommendations for Adolescents PA

Extraordinarily long-term attention has been devoted to PA recommendations for adolescents (37). However, it remains unclear to what extent global recommendations have mitigated deteriorating PA, and in what areas of a physically active lifestyle their promotion has been most effective and beneficial (16). Continental and regional variability in lifestyle is a significant inhibitor of globally recognized PA recommendations (38)—especially regarding children and adolescents—due to societal differences in the role of education, different educational systems, and curricula differences. This most likely explains why school PA recommendations are less frequently published, compared to general recommendations for PA for children and adolescents.

Recommendations published in the Healthy People Initiative and Physical Activity Guidelines for Americans (39) have had the greatest impact worldwide. There is an almost unanimous consensus that children and adolescents should engage in at least 60 min of PA daily (40–45), including MVPA focused on strengthening skeletal and muscular systems at least three times a week. Based on a study by Pate et al. (21), most of the 60 min of PA per day should be MVPA, and school PA should itself include at least 30 min of MVPA. According to our IPAQ-LF estimates, mean daily school PA is 62 min (268 METs-min/daily school time), 37 min of which are MVPA (185 METs-min/daily school time). Another equally important recommendation is that government institutions should ensure that schools provide PE programs in line with national standards of 225 min of PE per week in secondary schools for grades 9–12 (42).

Easy-to-understand PA indicators, such as minutes of PA or steps per day (or in particular segments of a day), are important for most adolescents. Regarding adults and the elderly, the universality of 10,000 steps per day is discussed worldwide and, despite many criticisms, its positive aspects have prevailed (46). The situation is more complicated for adolescents, due to their higher diversity of PA, especially in school and after-school activities. Despite frequent objections, recommendations of 10,000–11,700 steps per day for adolescents (47), or a simplified proposal for children and adolescents (of both sexes) of ≥11,500 steps per day (48), has been widely accepted in many countries.

In our study, we found that Czech adolescent boys and girls reached an average of 11,354 ± 3,606 and 10,799 ± 3,047 steps per day, respectively, while Polish adolescent boys and girls averaged 10,799 ± 3,692 and 10,130 ± 3,121 steps per day, respectively. Based on these findings, and previous studies in Central Europe (25, 31), we recommend 11,000 steps per day and 60 min of MVPA every day for both boys and girls. These results related to daily PA are also the starting point for creating PA recommendations in segments of school days.

### Recommendations for PA Before and After School

#### PA Before School

##### Background

The most significant type of PA before school for most adolescents is active transport (AT) to school. Declines in rates of AT to school have been observed in developed countries (49) and Central Europe (50). Although intervention studies have so far failed to increase AT to school significantly (51), it is desirable to keep investigating possibilities for promoting AT, particularly in less developed countries (e.g., Central and Eastern Europe), to avoid repeating the well-known causes of AT decline in more economically developed countries.

Before school PA is the most efficient use of available time and significantly contributes to increasing overall daily PA, mainly through AT (52). The distance from home to school is a crucial factor for AT among secondary school students (53). Approximately 0.84 miles was determined to be an acceptable distance for walking to school among Portuguese adolescents (54). Meanwhile, the US Healthy People 2020 plan advocates increasing the number of 5- to 15-years-olds who walk at least one mile to school (or cycle if the distance exceeds two miles).

##### Research findings

According to our pedometer-derived data, boys averaged 1,686 ± 910 steps (1,754 steps/h) and girls averaged 1,870 ± 972 steps (1,628 steps/h) in the before-school segment. Similar results were found from accelerometer monitoring, with boys averaging 1,465 ± 867 steps (1,451 ± 889 steps/h) and girls averaging 1,566 ± 899 steps (1,379 ± 1,001 steps/h). The average time for before-school PA was 37.2 ± 18.2 min for boys (MVPA ≥ 3 METs 9.5 ± 6.9 min and 9.6 ± 7.7 min/h; ≥ 60% HRmax 7.6 ± 15.8 min and 6.7 ± 10.5 min/h) and 40.2 ± 19.0 min for girls (MVPA ≥ 3 METs 8.9 ± 6.7 min and 7.9 ± 6.5 min/h; ≥ 60% HRmax 11.1 ± 14.9 min and 9.8 ± 11.3 min/h). Compared with other school-day segments, we also found the highest mean HR/min (boys: 96 ± 15; girls: 105 ± 15) during the before-school segment. Based on these findings, our proposed recommendations for PA in the before-school segment are at least 2,000 steps or 10 min of MVPA, mainly through AT with cycling or brisk walking.

##### Rationale

The proposed number of 2,000 steps before school was met by 29.3% of boys and 38.1% of girls, as measured by pedometers, and by 22% of boys and 25.4% of girls, as measured by accelerometers. The recommended 10 min of MVPA ≥ 3 METs was met by 37.2% of boys and 27.1% of girls, while 23% of boys and 39.6% of girls met the recommended 10 min of MVPA ≥ 60% HRmax. Observed sex differences highlighted the importance of multi-factor assessments of MVPA. Effective use of before-school time for PA was also confirmed by the ratio of PA time to total segment time; PA accounted for 58.1% of the before-school time for boys and 55.1% for girls.

#### PA After School

##### Background

After school PA is the most important part of daily PA on a school day (55); however, it is also the part most affected by educational and institutional environments (comprising sports and other leisure-time PA institutions), together with social and economic context. Despite their positive effects (56), previous after-school interventions to increase PA in children have scarcely yielded significant outcomes (57), and there is little evidence regarding which after-school settings increase PA among adolescents the most (58). Nonetheless, after-school programs have high potential (56) and, in coordination with school- and community-based sports clubs and institutions, they are indispensable for providing organized PA for children and adolescents.

##### Research findings

The PA of boys and girls, measured by pedometers, averaged 5,794 ± 3,389 steps (1,754 steps/h) and 6,188 ± 3,351 steps (1,628 steps/h), respectively, in the after-school segment. Furthermore, boys' and girls' PA as measured by accelerometers averaged 5,411 ± 3,513 steps (811 ± 494 steps/h) and 5,459 ± 3,122 steps (829 ± 454 steps/h), respectively. The average time of after-school PA was 153.7 ± 72.9 min for boys (22.8 ± 9.2 min/h; MVPA ≥ 3 METs 33.6 ± 26.4 min and 5.1 ± 3.9 min/h; MVPA ≥ 60% HRmax 37.0 ± 52.5 min and 5.5 ± 7.7 min/h) and 156.2 ± 65.3 min for girls (23.4 ± 7.7 min/h; MVPA ≥ 3 METs 30.0 ± 22.4 min and 4.6 ± 3.4 min/h; ≥ 60% HRmax 35.0 ± 48.2 and 5.4 ± 7.5 min/h). Mean HR/min was 90.5 ± 13.4 for boys and 93.8 ± 13.4 for girls. Based on these findings, our proposed recommendations for PA in the after-school segment are at least 6,000 steps or 30 min of MVPA, provided by AT from school and various organized and unorganized PA.

##### Rationale

The proposed number of 6,000 steps in the after-school segment was met by 41.5% of boys and 47.2% of girls, as measured by pedometers, and met by 33.4% of boys and 36.1% of girls, as measured by accelerometers. The recommended 30 min of MVPA ≥ 3 METs was met by 43.8% of boys and 40.4% of girls. Furthermore, PA accounted for 38.0 and 39.0% of the after-school time in boys and girls, respectively.

### School PA Recommendations

#### General School PA Recommendations in Summary and in Main Time Segments

##### Background

Most recommendations in national PA promotion programs are based on or complement the most widespread recommendations of Healthy People 2010, Healthy People 2020, and the 2008 Physical Activity Guidelines for Americans. They are formulated as calls for action (39, 59), such as:

- Increase the number of public and private schools that require daily PE for all students.
- Increase the number of adolescents who participate in daily PE.
- Increase the number of adolescents who are physically active for at least half of the PE allocated time.
- Encourage adolescents to participate in age-appropriate PA that is pleasant and diverse/varied.
- Increase the number of regular recess periods in schools.

In the context of educational systems' historical development (e.g., discretionary lessons, online PE, and lessons according to sports preferences), the recommendation and implementation of 225 min of PE per week still prevail in national educational curricula, despite the emergence of alternative approaches (60). It is also recommended that “schools should ensure that all children and adolescents participate in a minimum of 30 min of MVPA during the school day; this includes time spent being active in PE lessons,” [(60), p. 1,220] which is frequently promoted and generally accepted by educational institutions.

Based on PA monitoring of Czech and Polish adolescents, the following in-school PA recommendations were previously proposed: 3,000 steps/school time, 20 min of MVPA/school time (≥3 METs), 20 min of MVPA/school time (≥60% HRmax), and 25% PA/school time (22, 26). PA recommendations in segments of school days also began to be used based on the results of adolescents' weekly PA estimates from the IPAQ-LF questionnaire (Part 1—Job/school-related physical activity; 23). Despite questionnaire limitations (28), our school PA recommendations complement general PA recommendations as follows: ≥20 min of vigorous PA at school (≈360 METs-min) at least three times per week; ≥30 min of moderate PA at school (≈600 MET-min) at least five times per week; ≥30 min of walking at school (≈500 MET-min) at least five times per week; and (the most challenging recommendation) ≥60 min of any MVPA at least five times per week and ≥20 min of vigorous PA at least three times per week (≈ 1260 MET-min).

Despite extensive prior research on children's PA during recesses (61), little is known regarding the type of PA, its intensity, or benefits of PA-oriented recess and intervention programs to improve educational processes and increase overall daily PA in adolescents (62, 63). It is clear that appropriately inserting PA-oriented recess periods is a health-enhancing and cost-effective way to increase school PA (64). However, suitable indoor and outdoor school facilities are needed to facilitate PA during recess, thus addressing the prevalence of being overweight, obesity, and low levels of PA among secondary school students (62). It is important not to shorten recess; instead, the duration of the last lesson should be reduced (26).

Encouragingly, almost all countries recommend that PE be designated as a core subject (12). In most countries that recommend a minimum duration of PE time, the ratio of PE to overall school time is higher in primary than secondary education. Additionally, PE in secondary education represents 6–8% of total school time in Europe, ranging from 14% in France to 3–4% in Spain, Malta, and Turkey (15). On school days with physical education lessons (PEL), boys and girls not only achieve higher school PA but also higher overall PA (65). Regardless of lesson goals or content, at least 50% of each PEL should involve MVPA (60, 66). Moreover, students should be pushed to reach a significant physiological stress response nearing submaximal to maximal HR at least twice during each PEL, again independent of its focus (22).

##### Research findings

In our pedometer monitoring of school PA, boys averaged 3,653 ± 2,353 steps (580 ± 361 steps/h) and girls averaged 3,330 ± 1,969 steps (533 ± 309 steps/h). According to accelerometer monitoring, boys averaged 2,867 ± 1,849 steps (478 ± 296 steps/h) in school, while girls averaged 2,630 ± 1,659 steps (432 ± 243 steps/h). The mean time of PA (regardless of type) was 115.7 ± 47.2 min for boys (MVPA ≥ 3 METs 15.3 ± 14.0 min; MVPA ≥ 60% HRmax 19.2 ± 33.5 min) and 100.0 ± 43.4 min for girls (MVPA ≥ 3 METs 12.0 ± 11.3 min; MVPA ≥ 60% HRmax 21.1 ± 35.4 min). Mean HR/minute during school was 92.3 ± 12.3 in boys and 95.8 ± 11.4 in girls. Table 1 presents additional characteristics of school PA according to adolescent participation in PELs. As school PA with PELs was statistically significantly higher in both boys (>2,000 steps/school time and >14 min MVPA) and girls (>1,500 steps/school time and >11 min MVPA), compared to school PA without PELs, careful consideration should be given to the implementation of PA recommendations.

**Table 1:** School physical activity on days with and without a physical education lesson.

**Characteristics of school physical activity (measuring instrument)**	***n***	**School time**				***F***	***p***	***$\eta_{p}^{2}$***
		**Boys**		**Girls**				
		**with PEL**	**without PEL**	**with PEL**	**without PEL**			
		***M* (SD)**	***M* (SD)**	***M* (SD)**	***M* (SD)**			
Steps (pedometer)	2,103	5,538	2,950	4,496	2,930	154.93^a,b^	<0.001	0.181
		(2,507)	(1,853)	(1,850)	(1,846)			
Steps/h (pedometer)	2,103	857	477	714	471	140.86^a,b^	<0.001	0.168
		(388)	(290)	(296)	(289)			
Steps (accelerometer)	3,329	4,418	2,402	4,019	2,200	314.98^a.b^	<0.001	0.218
		(1,832)	(1,582)	(1,753)	(1,386)			
Steps/h (accelerometer)	3,329	730	402	640	368	333.88^a,b^	<0.001	0.228
		(281)	(256)	(252)	(201)			
PA-min (accelerometer)	3,329	145.8	106.7	134.5	89.3	265.31^a,b^	<0.001	0.190
		(44.8)	(44.1)	(39.6)	(38.7)			
PA-min/h (accelerometer)	3,329	24.2	18.4	21.5	15.1	280.44^a,b^	<0.001	0.199
		(6.7)	(6.6)	(5.5)	(5.7)			
MVPA-min (accelerometer)	3,329	26.2	12.0	20.8	9.4	273.40^a,b^	<0.001	0.195
		(15.4)	(11.8)	(12.7)	(9.3)			
MVPA-min/h (accelerometer)	3,329	4.3	2.0	3.3	1.5	285.19^a,b^	<0.001	0.202
		(2.4)	(1.9)	(2.0)	(1.4)			

*PEL, physical education lesson; M, Mean; SD, Standard deviation; F, value of ANOVA; p, level of significance; $\eta_{\text{p}}^{2}$, effect size coefficient; ^a^significant difference between boys with and without PELs; ^b^significant difference between girls with and without PELs*.

On average, boys reached 1,229 ± 1,298 steps (278 ± 285 steps/h) and girls reached 1,094 ± 1,135 steps (242 ± 243 steps/h) during non-PE school lessons. From the first lesson (1L) to the sixth lesson (6L) in school schedules (six lessons is a common school day in vocational schools in the Czech Republic and Poland), boys averaged 174 ± 465 steps in 1L, 182 ± 428 steps in 2L, 151 ± 392 steps in 3L, 133 ± 217 steps in 4L, 170 ± 281 steps in 5L, and 271 ± 429 steps in 6L, while girls averaged 131 ± 307 steps in 1L, 118 ± 240 steps in 2L, 132 ± 322 steps in 3L, 119 ± 225 steps in 4L, 157 ± 315 steps in 5L, and 238 ± 412 steps in 6L. Aggregated across all lessons during school time (excluding PELs), boys averaged 1,216 ± 1,325 steps (267 ± 283 steps/h) and girls averaged 1,088 ± 1,132 steps (236 ± 237 steps/h). Further, it is concerning that we observed HR ≥ 85% HRmax during lessons in 9.9% of boys and 13.0% of girls.

According to the pedometer monitoring of PELs, boys averaged 2,554 ± 1,108 steps/45 min, and girls averaged 1,839 ± 828 steps/45 min. Boys and girls monitored by accelerometer averaged 1,894 ± 1,144 steps (1,604 ± 784 steps/45 min) and 1,585 ± 843 steps (1,308 ± 624 steps/45 min), respectively. The average PA time in PELs was 35.0 ± 7.2 min/45 min for boys (MVPA ≥ 3 METs 11.3 ± 7.2/45 min; MVPA ≥ 60% HRmax 20.1 ± 14.6/45 min) and 33.3 ± 7.02 min for girls (MVPA ≥ 3 METs 8.1 ± 5.5/45 min; MVPA ≥ 60% HRmax 18.3 ± 13.4/45 min). Based on these findings, our proposed PA recommendations for in-school segments are for students to reach at least 3,000 steps (averaging at least 500 steps/h, lesson, and recess) and at least 20 min of MVPA during school. Furthermore, it should be ensured that there is at least one significant physiological response to vigorous PA intensity (at submaximal to maximal HR). School PA should account for at least 25% of total school time, and 50% of their recess should be devoted to PA. Recess should last for at least 25% of the total time of lessons. In each PEL, students should strive to achieve at least 2,000 steps/45 min and at least 20 min of MVPA/45 min, with more than 50% of PEL time devoted to PA, and at least two physiological responses at submaximal to maximal HR.

##### Rationale for PA during total time spent in school

**Figure 1:**
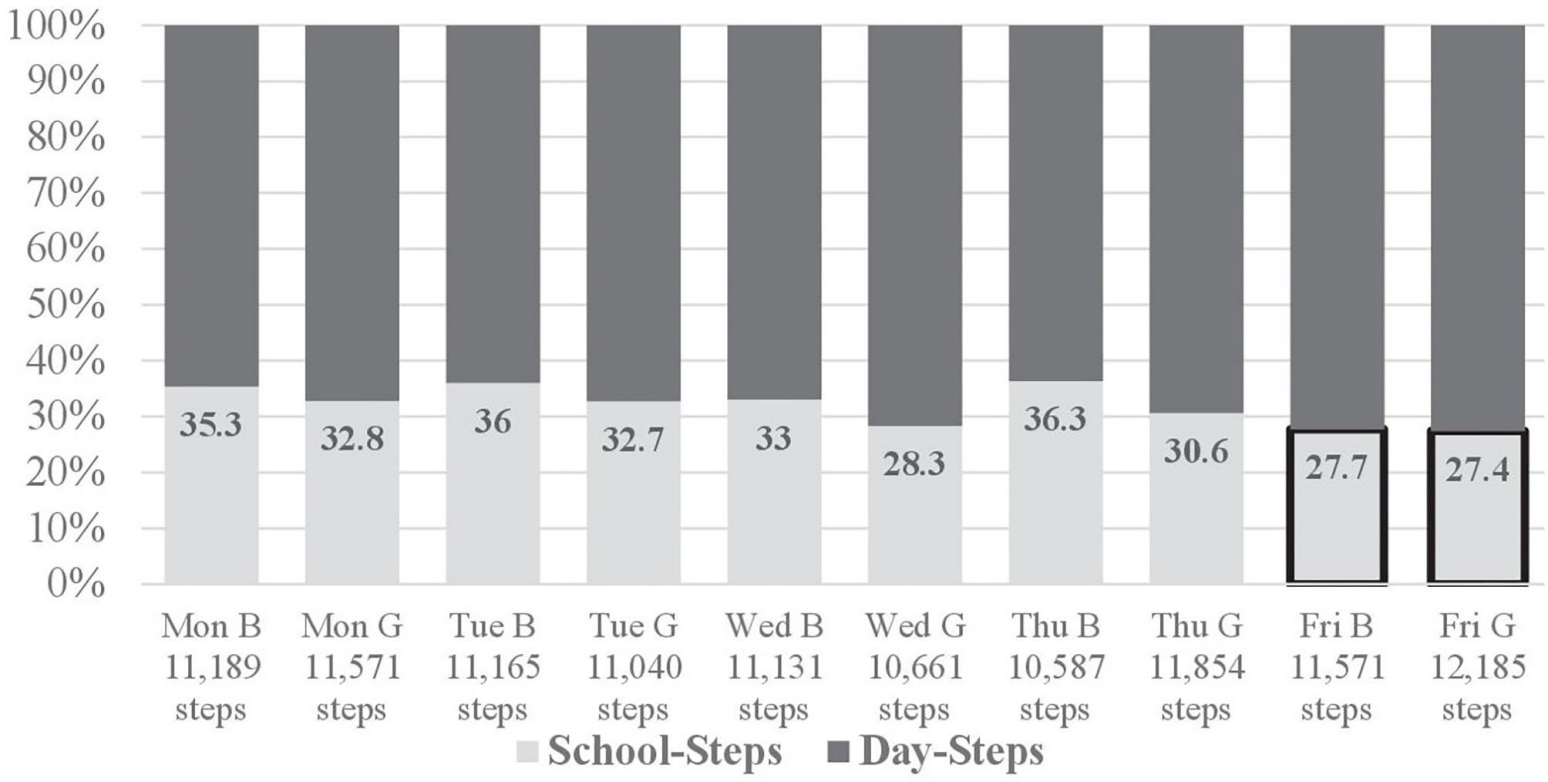
Ratio of boys' (B) and girls' (G) step counts in school to total daily step counts on weekdays (monitored by pedometers).

The proposed recommendation of 3,000 steps during school time was met by 50.4% of boys and 49.8% of girls. Similarly, 49.1% of boys and 46.6% of girls met the recommendation of 500 steps per hour. The ratios of boys' and girls' step count in school to their total daily step counts on weekdays are shown in Figure 1.

The recommendation of 3,000 steps in school, based on accelerometry derived PA monitoring, was met by 37.9% of boys and 32.6% of girls. Further, 38.1% of boys and 31.7% of girls met the recommendation of 500 steps/h. The recommended 20 min of MVPA ≥ 3 METs was met by 29.1% of boys and 19.1% of girls, while the MVPA recommendation ≥ 60% HRmax was met by 27.2% of boys and 30.1% of girls. PA accounted for 32.4% and 27.8% of overall school time for boys and girls, respectively. Additionally, according to responses to the IPAQ-LF, school PA comprised 28.8% of overall 5-days PA in boys and 27.1% in girls.

Among the pedometer-measured sample, the proposed 3,000 steps in school were met by 85.0% of boys and 79.7% of girls on days with PELs, compared to 37.5 and 39.6%, respectively, on days without PELs. The equivalent values in the accelerometer-measured sample were 77.1% of boys and 68.3% of girls on PEL days, compared to 26.1 and 21.6%, respectively, on non-PEL days. In the pedometer-measured sample, the recommendation of 500 steps/h was met by 81.8% of boys and 77.6% of girls on PEL days, compared to 37.0 and 36.0%, respectively, on non-PEL days. The equivalent values in the accelerometer-measured sample were 80.2% of boys and 64.4% of girls on PEL days, compared to 25.5 and 21.5%, respectively, on non-PEL days. The recommended 20 min of MVPA at school was met by 41.3% of boys and 58.7% of girls on PEL days, compared to 19.5 and 11.7%, respectively, on non-PEL days.

In total, on school days without PELs, 65.0% of boys and 44.5% of girls met the recommendation that PA should represent 25% of school time. However, 93.9% of boys and 91.0% of girls met this recommendation on PEL days. These values comprise all forms of PA. It is important to note that low-intensity PA and short bouts of MVPA have significant health benefits in the school environment (67).

School PA recommendations apply regardless of the type of school or the presence of PELs in the daily program. Therefore, demands will be higher on days with PELs and in schools oriented toward practical lessons.

##### Rationale for PA during recesses

Boys were physically active for 54.6 ± 16.1% of recess time, compared to 50.1 ± 14.5% for girls. However, MVPA ≥ 3 METs accounted for only 6.2 ± 6.3% of total recess time for boys and 4.8 ± 4.9% for girls, and MVPA ≥ 60% HRmax accounted for 8.5 ± 11.6% of total recess time for boys and 8.9 ± 6.4% for girls. Both the intensity and duration of PA during recesses were found to be low, with boys averaging only 1,056 ± 524 steps per hour and girls averaging 1,023 ± 485 steps per hour in a total of 60 min of recess each day. These results confirm that longer cumulative recess time increases school PA in boys and girls but cannot replace participation in PELs (22).

##### Rationale for PA in physical education lessons

PA in PELs represented 77.8 ± 16.1% of total PEL time in boys; however, MVPA ≥ 3 METs accounted for only 25.2 ± 16.1% and MVPA ≥ 60% HRmax for only 44.7 ± 32.4% of total PEL time. In girls, PA represented 74.0 ± 15.5% of total PEL time; however, MVPA ≥ 3 METs accounted for only 18.0 ± 12.2% and MVPA ≥ 60% HRmax for only 40.6 ± 29.7% of total PEL time. In many cases, PELs play a significant role in meeting general PA recommendations, and further improvement is needed to promote adherence to lifelong PA.

#### Model of PA Recommendations for Segments of the School Day

**Figure 2:**
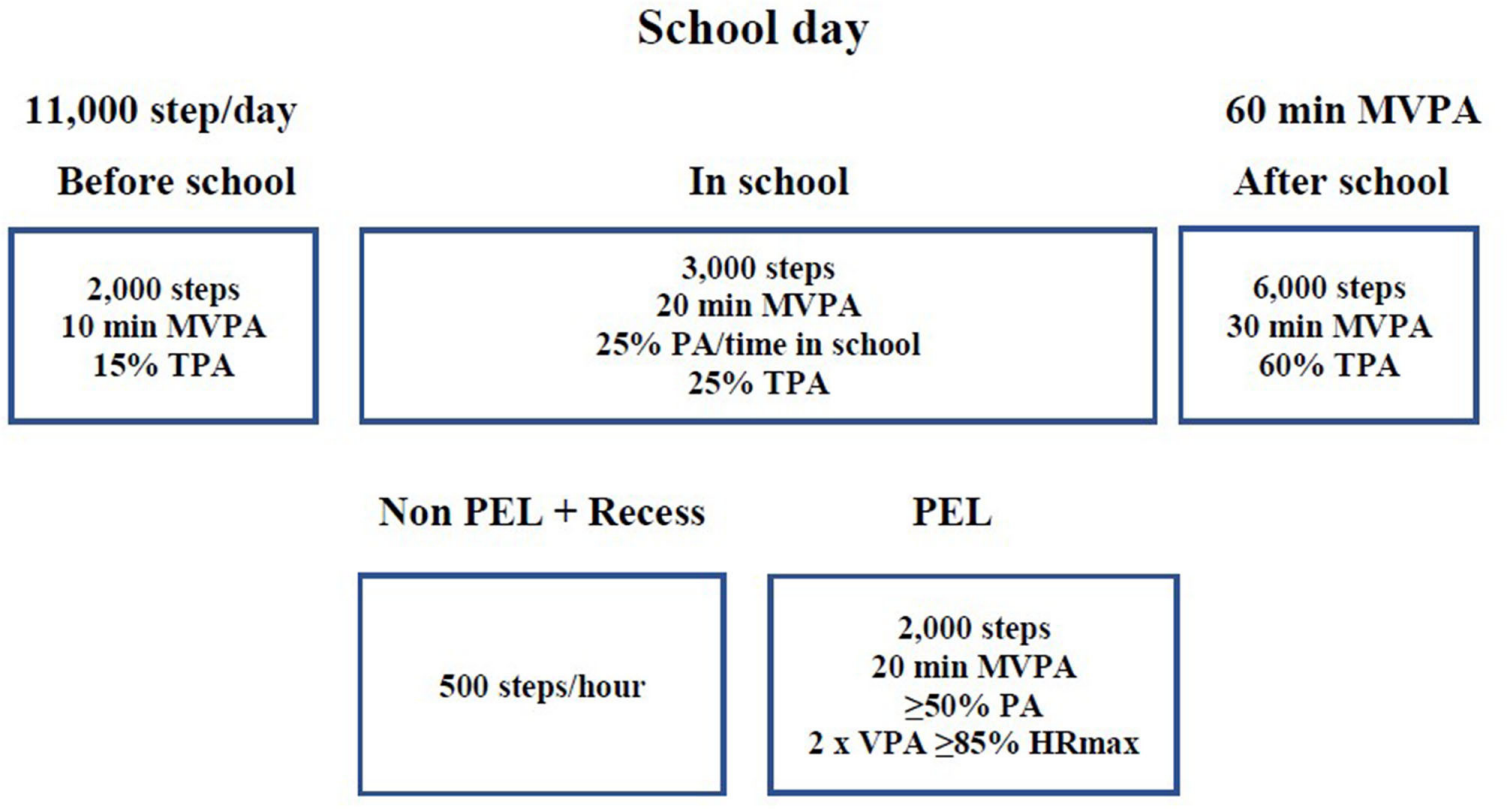
Model of recommendations for school physical activity.

Figure 2 depicts a simplified guide for adhering to minimum PA recommendations in the main segments of a school day, emphasizing a substitution approach under specific conditions. It includes the following indicators: number of steps, MVPA minutes, each segment's share of total PA (TPA), and submaximal and maximal HR (HRmax).

## Discussion

PA recommendations in segments of school days should be evidence-based with respect to PA, which will likely lead to simplification of universal indicators primarily due to health and educational differences, alongside political, socioeconomic, and demographic differences. Universal PA recommendations in segments of school days should be an enduring guideline, especially to motivate at-risk adolescents and support health and physical literacy. It should also link the school schedule with a normal lifestyle, even for weekends and school holidays. To emphasize increasing adolescents' PA through applying a comprehensive multi-sector strategy (60), PA recommendations in segments of school days need a multi-sector basis and multi-factor indicators (e.g., time, intensity, frequency, and volume of PA) to enable alternative approaches to its use. Thus, the proposed PA recommendations in segments of school days Model is based on existing evidence-based findings on school PA in Central European countries (22, 26). There remains a lack of high-quality intervention studies on school PA in these countries, compared to Western European countries (68). Amidst significant educational reforms in Central Europe, the issue of PE has received insufficient attention, with the sole possible exception of Hungary (69). Therefore, given their common historical roots, our suggested recommendations for the Czech Republic and Poland are also potentially applicable to Slovakia, Hungary, and other Eastern European countries.

The model of PA recommendations in segments of school days could support the formation of evidence-based strategies for developing school PA, as well as facilitating and improving means to track the impact of such strategies on health and educational outcomes, in line with the guiding principles and priorities of (18) PA strategy. Proposed recommendations for school PA should support positive changes in the organization of educational processes in secondary schools. Enforcing the 500 steps per hour recommendation is especially important because it could promote more efficient use of classroom-based PA and better mitigate educational stress. Integrating PA into non-PE lessons may increase students' overall PA, improve their attention and working time, and positively influence academic outcomes (70, 71). The identified association between the number of steps per hour and self-reported stress levels in periods of physical inactivity, highlight the need to compensate for educational workloads through PA and the importance of integrating PA into classroom-based lessons.

School recess can make a significant contribution to meeting the 500 steps per hour recommendation. In our research, recess accounted for only 15% of total school time each day. The recommended 10-min recess periods with one longer 20-min recess, excluding any other PA- or health-oriented breaks or lunch breaks, should represent at least 20% of the total in-school time. In earlier research, 41% of boys met the recommended 500 steps per hour when given a longer total recess time (≥60 min), compared with 26% of boys with a shorter recess time (<60 min); the equivalent values for girls were 42 and 23%, respectively (53). These findings emphasize the importance of increasing total recess time; however, they also show that longer recess time cannot replace students' participation in PELs, given the documented mean steps in the latter. It is encouraging that we found positive associations between meeting the PA recommendations and a lower incidence of depressive symptoms and academic stress in adolescents (27, 72).

Pedometer-measured steps in PELs (boys: 2,554 steps/45 min; girls 1,839 steps/45 min) corresponded to unpublished results we collected during teacher-student training. Within these “training lessons,” boys (*n* = 1,858) averaged 2,311 ± 871 steps/45 min and girls (*n* = 2,234) averaged 1,852 ± 717 steps/45 min. Similar results in PELs were found by Culpepper and Killion (73), in which boys averaged 2,454 ± 838 steps/45 min, and girls averaged 1,820 ± 750 steps/45 min.

We know that a substantial percentage of recommended children and adolescent PA can be provided through a comprehensive school PA program (74, 75). Thus, PA recommendations in segments of school days should become an integral part of these programs, to encourage changes toward a healthy school lifestyle, facilitate the adoption of (necessary) healthy work habits, and raise awareness of how mental stress can be mitigated through adequate PA. Implementing PA recommendations in segments of school days into comprehensive school PA programs could also provide a good basis for finding complementary forms of PE. Support for comprehensive school PA program development should form part of national PA policies or promotion plans, although many countries still fail to optimize their use (42).

PA recommendations in segments of school days should be an integral part of comprehensive school PA programs, as it can assure a significant part of daily recommended PA (70). PA recommendations in segments of school days also have potential economic benefits (76). Application of PA recommendations to the main segments of the school day can support the determination of the economic and social effects of adolescents at schools' health. School PA has significant social potential, and therefore the prevalence of PA recommendations in segments of school day could support the social health of adolescents within the school. It is confirmed that adolescents meeting the PA recommendations are also more likely to have better subjective well-being than others (27). Future research should focus on obtaining evidence in support of adolescent PA by applying PA recommendations in segments of school days to schools. Furthermore, it should focus on exploring the positive and negative aspects of PA recommendations in segments of school days enforcement in comprehensive school PA programs, not only among adolescents but also among teachers and school administrators.

### Strengths and Limitations

To the best of our knowledge, this was the first study dealing with the comprehensive promotion of school PA recommendations for specific segments of school days. This study's strength lies in its long-term comprehensive analysis of evidence and experience-based findings on school PA across different types of secondary schools in the Czech Republic and Poland, with a large sample of adolescents. Additionally, it combines subjective PA estimates with PA monitoring by pedometers and accelerometers, yielding a uniform dataset processed and analyzed through the web application Indares.com. It was essential, to the greatest possible extent, to keep natural and customary conditions in all stages of our research conducted in schools.

However, our PA recommendations are limited in application to Central Europe and countries with similar geographic, socioeconomic, and educational conditions. As we do not have enough evidence to determine prospective PA recommendations based on the current compliance rate, the starting point for determining the presented PA recommendations in this study was to meet the recommendations for 30–50% of participants. The setting of minimum recommendations was mainly based on the level and trends of PA, but also on the experience with the presentation of results in schools in direct contact with participants. Our school PA results on days with PELs were influenced by the goals and content of PELs, which were solely determined by PE teachers (without our input). The major limitation was that the suggested recommendations for PA in segments of school days were determined based only on the status of, and trends in, adolescent PA and the practical experience gained by implementing research results into school practice. Future research should focus on the verification of proposed PA recommendations in segments of school days in different educational systems. Furthermore, to establish under which circumstances PA recommendations in particular segments of the day may support and increase adolescents' daily PA.

## Conclusions

The results of the trends in PA of Czech and Polish adolescents enabled the study to propose a comprehensive model of recommendations for adolescent PA in individual segments of the school day. The implementation of these partial PA recommendations in school practice can support increased PA in some adolescent groups and positive changes in the school lifestyle. As part of the acquisition of physical literacy, these simplified PA recommendations should also be placed in the context of the risks of unilateral PA, the development of motor skills and physical fitness. These findings will need further investigation in the context of traditional daily and weekly PA recommendations. It will also be very important in school practice to respect the rapid development and popularity of technologies used in PA monitoring to adopt recommendations for adolescent PA in segments of the school day. Recommendations on adolescents PA in school day segments should also become a part of the design and innovation of school PA programs.

## Data Availability Statement

The datasets generated for this study are available on request to the corresponding author.

## Ethics Statement

The studies involving human participants were reviewed and approved by the Ethics Committee of Human Research of the Faculty of Physical Culture, Palacký University, in Olomouc (no. 24/2012) and the Jerzy Kukuczka Academy of Physical Education, in Katowice (no. 2/2008). Written informed consent to participate in this study was provided by the participants' legal guardian/next of kin.

## Author Contributions

KF, DG, and JM collected data, secured funding, drafted, and conceptualized the manuscript. AM and TC reviewed and edited the study. All authors contributed to the article and approved the submitted version.

## Conflict of Interest

The authors declare that the research was conducted in the absence of any commercial or financial relationships that could be construed as a potential conflict of interest.
